# Trimethylamine-N-oxide as One Hypothetical Link for the Relationship between Intestinal Microbiota and Cancer - Where We Are and Where Shall We Go?

**DOI:** 10.7150/jca.31737

**Published:** 2019-10-08

**Authors:** Carmen Wing Han Chan, Bernard Man Hin Law, Mary Miu Yee Waye, Judy Yuet Wa Chan, Winnie Kwok Wei So, Ka Ming Chow

**Affiliations:** The Nethersole School of Nursing, The Chinese University of Hong Kong, Shatin, the New Territories, Hong Kong, China.

**Keywords:** cancer, trimethylamine, trimethylamine-N-oxide, intestinal microbiota

## Abstract

Previous epidemiological studies had provided evidence for a link between the microbial dysbiosis and cancer, particularly colorectal cancer (CRC), yet the molecular basis of this link remains elusive. Recently, the association between plasma levels of trimethylamine-N-oxide (TMAO), an oxidised form of trimethylamine (TMA), and risks of various cancers was demonstrated. The discovery could potentially provide an alternative explanation for the aforementioned link, as TMA production is attributed to intestinal bacteria. Current evidence suggests that inflammation could be a potential molecular mechanism to explain the link between TMAO and cancer, although other mechanisms such as oxidative stress, DNA damage and disruption in protein folding might also play a role. This mini-review article first provides an overview of the current evidence for the association between TMAO and certain cancer types, and the potential mechanisms that could explain their association. Thereafter, the direction of further research on the connection between the intestinal microbiota, TMAO and cancer is suggested.

## Introduction

In the past decades, cancer has become one of the most prevalent non-communicable diseases worldwide, with more than 14 million people worldwide having developed cancer in 2012 1. Emerging studies over the past years suggested that microbial dysbiosis, a pathogenic change in the composition of the intestinal microbiota, is one of the potential factors that are associated with increased risk of certain cancers, in particular colorectal cancer (CRC) 2. Indeed, numerous studies had reported a change in the intestinal abundance of certain bacterial genera or species in CRC patients and animal models 3. Further, possible mechanisms were proposed as to how microbial dysbiosis may lead to carcinogenesis. These mechanisms include intestinal inflammation and genotoxin-induced DNA damage of intestinal cells 4, 5. A more detailed description on the potential mechanism in how the intestinal microbiota is linked to various cancer types was conducted by Sheflin *et al* 6, and how certain bacterial species can potentially lead to development of cancer was reviewed by Hullar *et al* 7.

In addition to the aforementioned possible mechanisms on how intestinal microbiota is related to cancer, some recent studies suggest that trimethylamine-N-oxide (TMAO), an oxidised metabolite of trimethylamine (TMA), could also perhaps be a potential candidate that may provide a link for this relationship. In this mini-review, we will first describe how TMAO is produced by the intestinal bacteria and the data that reveal the association between TMAO and increased cancer risks, highlighting the benefits of further research into this association. We will then discuss the possible further work that would provide evidence and facilitate our understanding on this association. This article therefore serves to make a case for further exploration of this relationship, which would provide further insights into how diet and intestinal microbiota is associated with cancer risks.

### TMAO: The Source

TMAO generation in humans involves a two-step sequential mechanism from precursor molecules such as choline and L-carnitine via TMA. These precursor molecules, which were shown to be present abundantly in red meat, eggs, milk and certain fish products including salmon 8, 9, would first be converted to TMA by certain TMA-producing bacteria in the gut microbiome. Thereafter, TMA would be absorbed through the intestinal wall and transported to the liver, where it would be metabolised into TMAO through oxygenation, a process catalysed by flavin-containing monooxygenase 3 (FMO3) 10.

Previous studies provided several lines of evidence that the intestinal microbiota plays a significant role in TMAO production. Koeth *et al* 11 demonstrated that human subjects who had undergone one-week treatment with antibiotics, for the purpose of suppressing their intestinal microbiota, had a significantly lower level of plasma and urinary TMAO following a carnitine challenge test, compared to untreated controls. Likewise, subjects who received a phosphatidylcholine challenge also exhibited a reduced plasma TMAO levels upon administration of antibiotics, and such effect was abolished after the removal of the administered antibiotics 12. Such phenomenon was also observed in a mouse model 13. Further, Romano *et al* 14 had recently revealed a number of intestinal bacterial species that are able to produce TMA following an *in vitro* incubation in mice gut medium supplemented with choline. These TMA-producing bacteria largely belong to the *Firmicutes* and *Proteobacteria* phyla. One observation of note is that none of the bacterial species screened for TMA production in the Romano *et al* study belong to *Bacteroidetes*, one of the major bacterial phyla present in the human intestine. Consistent with this observation, previous studies demonstrated that the enzymes required for the conversion of choline and L-carnitine to TMA are present in *Firmicutes* and *Proteobacteria*, but these enzymes are absent in *Bacteroidetes* 15, 16. These observations therefore suggest that the level of TMA production is not only dependent on the amount of choline and L-carnitine intake, but could also perhaps be modulated by changes in the composition of the intestinal microbiota, such as the ratio of the abundance of *Firmicutes* and *Bacteroidetes*, the two major bacterial phyla present in the human intestine. In line with this hypothesis, a recent study demonstrated that individuals who produced a higher level of TMAO (high TMAO producers) following the intake of high-choline or high-carnitine diet possessed a higher *Firmicutes* to *Bacteroidetes* (F/B) ratio in their intestinal microbiota than the low TMAO producers 17. Nevertheless, further human and animal studies are necessary to confirm this hypothesis.

Of note, however, changes in TMAO levels are not only solely dependent on the action of intestinal bacteria, but could also be contributed by dietary intake. Indeed, previous studies had indicated that fish intake among human subjects would lead to a significant increase in their circulatory TMAO levels 17, 18, and positive association was observed between fish consumption and plasma TMAO levels in humans 19. As indicated by Landfald et al. 20, free TMAO is present in large quantities in a variety of seafood, including fish, and it is plausible that such observed increase in circulatory TMAO levels after fish intake could be attributed to the intake of the free TMAO present in fish. Nevertheless, current findings on the association between fish consumption and TMAO levels are inconsistent between studies, as one study reported a lack of an association between the two aforementioned parameters 21. Overall, increase in TMAO levels can be attributed to two sources: 1) TMA production from precursor molecules by the action of intestinal bacteria and subsequent oxidation in liver; and 2) dietary intake of TMAO-rich foods, although further studies are needed to confirm the contribution of the latter to increase in TMAO levels.

### Potential Relationship between TMAO and Cancer: The Evidence

With the understanding of the relationship between intestinal microbiota and carcinogenesis, particularly CRC, research efforts had started to focus on whether intestinal microbiota-dependent metabolites (including TMAO) could also be linked to cancer. One of the first studies to investigate this link was published in 2014, where Bae *et al* 22 showed that plasma TMAO levels are positively correlated with CRC risks among women in the United States. In the study, subjects in the highest quartile of plasma TMAO concentration were found to be at 3.4 times greater risk of rectal cancer than those in the lowest quartile of plasma TMAO concentration. A recent study also demonstrated an increased serum TMAO level among CRC patients, compared to healthy controls, rendering TMAO a potential prognostic marker for CRC 23. These data therefore suggest that TMAO could either directly or indirectly lead to CRC. Interestingly however, a recent study showed that no obvious associations exist between serum TMAO levels and rectal cancer risks among Finnish men 24. Nevertheless, another study demonstrated that TMAO could serve as an intermediate that links the metabolic activities of the intestinal microbiota and CRC pathogenesis 25, thereby providing further supporting evidence for a role of TMAO in CRC risks. The authors showed that there are certain genetic pathways that are associated with both TMAO and CRC carcinogenesis. These pathways include cell cycle progression and Wnt signalling. The sharing of these pathways by both TMAO and CRC implies that these pathways could be involved in the link between TMAO and CRC. It is therefore tempting to speculate that increased TMAO levels could perhaps modulate the activity of these pathways, although this hypothesis requires confirmation through further studies.

In addition to CRC, published data also suggest that TMAO could be related to the increased risk of other cancers. Metabolomic profiling analysis of prostate cancer patients and healthy controls in a recent cohort study in Finland revealed a positive correlation of an increased level of plasma TMAO with aggressive prostate cancer risk 26. This correlation was further supported by a previous finding that increased choline intake, which subsequently leads to increased TMA and TMAO production, is also associated with increased risk of lethal prostate cancer 27. Further, proton nuclear magnetic resonance (NMR) spectroscopic data revealed that patients with oral squamous cell carcinoma has a higher seral level of TMAO compared to healthy controls 28. These studies therefore suggest that TMA production by intestinal microbiota, and the subsequent increase in plasma TMAO levels, not only may modify CRC risks, but also the risk of other cancer types such as prostate cancer and oral cancer.

Nevertheless, one indeed may argue against the positive relationship between TMAO and cancer with the evidence that increased fish intake, which was shown to reduce cancer risks in a meta-analysis 29, would increase circulatory TMAO levels 17, 18. However, despite the increased intake of free TMAO, fish consumption is also associated with the intake of certain health-promoting compounds shown to be potentially protective against cancer. For example, fish oil present in fish contains an abundant amount of omega-3 polyunsaturated fatty acids, such as docosahexaenoic acid and eicosapentaenoic acid, and these were previously shown to exhibit chemo preventive effect of CRC via mechanisms such as downregulating the expression of pro-cancerous genes including pro-inflammatory cytokines 30. It is possible that the cancer chemo preventive effect of fish oil intake would outweigh the detrimental effect of increased TMAO levels in the promotion of cancer, making increased fish intake being protective against cancer despite its promoting effect on circulatory TMAO levels. Notably, however, further studies may be needed for confirmation of this hypothesis.

Taken together, data from previous studies have contributed evidence to a previously unappreciated relationship between TMAO and carcinogenesis, even though a recent study demonstrated no relationship between TMAO levels and CRC risks 24. While we cannot rule out the possible effect of gender and ethnic differences on this relationship, the observed discrepancies could perhaps be explained by the inclusion of different covariates in the multivariate logistic regression analyses performed in these two studies. For example, after adjusting for factors such as education level and energy intake of subjects, TMAO was found not to be associated with rectal cancer risk 24, as did when these covariates were not included in the regression analysis 22. Furthermore, other factors, such as kidney function, could exhibit confounding effects on the gut microbiome that produce metabolites that could interact with TMAO, and flavin-containing monooxygenase 3 (FMO3) genotype 31. Moreover, as suggested by Ufnal and Pham 32, plasma TMAO levels can be affected by factors such as the permeability of gut-blood barrier, which has a function in regulating the entry of molecules from the gut into the bloodstream. Therefore, assessment of the association between plasma TMAO levels and disease risk may need to take into account of gut-blood barrier permeability, assessing this parameter as a confounding factor, potentially via the double sugar test 33. Indeed, a recent study observed a higher TMA level in the blood in hypertensive rats which were characterised by an increased permeability of the gut-blood barrier to TMA, resulting in more TMA being delivered to the liver where the TMA is oxidised to TMAO 34. The authors also suggested that it is the permeability of the gut-blood barrier that may modulate disease risk, rather than plasma TMAO levels. Therefore, the discrepancies in the findings between studies reporting associations between TMAO levels and cancer risks could also be attributed to differences in gut-blood permeability between individuals. Nevertheless, with the finding that TMAO is involved in a number of cancer-related cellular pathways 25, it is still worthwhile to explore further on the relationship between TMAO and cancer.

### Potential Relationship between TMAO and Cancer: Possible Mechanisms

With increasing evidence suggesting the potential association between TMAO levels and cancer risk through observational association studies described above, research had recently started directing towards the exploration of the possible mechanisms on how TMAO could contribute to carcinogenesis.

Currently, several lines of evidence have suggested that inflammation could be a possible factor that provides a link between TMAO and cancer. Indeed, the causal relationship between chronic inflammation and carcinogenesis, including CRC, is already well-established 35, 36. However, recent evidence has suggested that TMAO could play a role in the inflammatory process. For example, TMAO was found to synergise the pro-inflammatory effects of *H. pylori* infections on gastric epithelial cells, through the increase in the expression level of pro-inflammatory genes including interleukin-6 (IL-6) and chemokine ligands 37, which were shown to play roles in cancer progression 38-41. This finding therefore suggests a potential link between TMAO and gastric cancer via the inflammatory process. Consistent with this, Yue *et al* also demonstrated that TMAO can trigger the activation of the nod-like receptor family pyrin domain containing 3 (NLRP3) inflammasome 42, 43, which was suggested to be implicated in the growth and/or metastasis of a variety of cancers including head and neck cancer, oral cancer, lung cancer, prostate cancer and colorectal cancer 44-46. Moreover, association studies had provided further support for a link between TMAO and inflammation. Serum level of TMAO was shown to be positively correlated with the level of certain pro-inflammatory mediators including tumour necrosis factor-alpha (TNF-α) and IL-6 among diabetic patients with chronic kidney disease 47. An earlier study within a German population also revealed that plasma TMAO concentrations exhibit a positive association with plasma TNF-α levels 21. Since both TNF-α and IL-6 are implicated in carcinogenesis through the induction of chronic inflammation 48, these data therefore provide further evidence that inflammation could be a likely factor for the contribution of TMAO to carcinogenesis. Further research on this link is recommended to delineate the mechanism on how TMAO is linked to cancer via inflammation induction.

The role of TMAO in exacerbating inflammation can further be demonstrated by the reported potential association between TMAO and diseases such as cardiovascular disease, atherosclerosis and chronic kidney disease 49-51, which, like cancer, were found to be associated with the occurrence of pro-inflammatory events 52-54. Indeed, a recent systematic review on 11 cohort studies demonstrated a positive association between circulatory TMAO levels and cardiovascular disease risks in humans 55, and a cohort study also showed that plasma TMAO levels were higher among patients with chronic kidney disease 56. TMAO was found to promote vascular inflammation in mice via activation of nuclear factor-kappa B and the subsequent increase in the expression of pro-inflammatory mediators 57. Further, renal dysfunction, which occurs in chronic kidney disease, in mice was found to be contributed by increased TMAO levels and the subsequent induction of inflammation 58. These findings add further weight to the contributory role of TMAO in inflammation. With inflammation known to be implicated in carcinogenesis 48, these findings could strengthen the argument that TMAO may contribute to carcinogenesis through inflammatory pathways. Nevertheless, it should be pointed out that recent studies have also revealed an indirect protective effect of TMAO against cardiovascular disease. For example, low-dose chronic treatment of hypertensive rats with TMAO was shown to help improve their heart function and reduce cardiac fibrosis, thereby protecting these rats against hypertension 59. Further, L-carnitine treatment was also found to reduce the severity of aortic lesions in mice via an increase in TMAO levels, demonstrating the protective effect of TMAO against atherosclerosis 60. Notably, both hypertension and atherosclerosis are known risk factors of cardiovascular disease 61, 62.

Oxidative stress might also be one of the factors linking TMAO and cancer. Oxidative stress was previously suggested to contribute to cancer via the activation of inflammation 63, 64. Recently, studies showed that TMAO could be implicated in oxidative stress. Increased level of circulating TMAO was shown to induce superoxide production, a reactive oxygen species (ROS) linked to oxidative stress 65. In an *in vitro* assay, TMAO was also shown to stimulate the production of ROS in cells 43. Nevertheless, more studies are needed in order to confirm the association between TMAO, oxidative stress and cancer.

In addition, other studies have also contributed evidence that helps suggest the possible molecular mechanisms in how TMAO could be linked to cancer. For example, TMAO was suggested to be involved in molecular pathways that lead to the generation of N-nitroso compounds 66, which were known to induce DNA damage, an event that can contribute to carcinogenesis 67. This suggests a potential involvement of DNA damage in TMAO-contributed carcinogenesis. Furthermore, TMAO could lead to the loss-of-function of alpha-casein 68, a chaperone protein previously found to function as a tumour suppressor that helps prevent breast cancer 69, implying that disrupted quality control of protein folding could also be involved in TMAO-contributed carcinogenesis.

Overall, inflammation appears to be a promising candidate that could provide a link between TMAO and carcinogenesis. Other potential mechanisms, including oxidative stress, might also be involved, despite the need for further validation through association and molecular studies.

### TMAO and Cancer: The Cause or the Consequence?

Despite the promising evidence that TMAO could be implicated in an increase in cancer risks, the role of TMAO in contributing to carcinogenesis remains controversial. Although previous studies did observe a higher level of plasma/seral TMAO among cancer patients, it remains unclear whether such increase in TMAO levels is a cause or a consequence of cancer. Currently, there is a scarcity of studies that explore whether alterations in TMAO levels would have affected certain molecular pathways or gene expression profiles directly implicated in carcinogenesis. As a result, it is difficult to determine whether TMAO is really causative of cancer or a sheer marker for tumour formation and/or progression. Indeed, the elusiveness on whether TMAO is a molecular contributor to disease development or it is a sheer marker for a disease-associated condition was previously suggested 31. After all, previous studies also showed that TMAO could potentially be implicated in the inhibition of molecular pathways suggested to be involved in cancer progression. For example, TMAO was shown to play a role in the reduction of endoplasmic reticulum (ER) stress 70, 71, a condition previously suggested to be implicated in cancer cell proliferation and survival, which leads to tumour progression 72-74. Nevertheless, the mechanisms on how TMAO can inhibit ER stress remain to be dissected, although its role in attenuating unfolded protein response, a cellular response that helps ameliorate ER stress, was previously suggested 70. Such observations highlight the current uncertainty in how TMAO is implicated in cancer.

In light of such contrasting findings, it is of interest that the role of TMAO in carcinogenesis be established. Its potential contribution to tumour formation and progression should be explored. This would help establish whether TMAO can serve as a molecular target for dietary interventions dedicated for cancer prevention, thereby opening up further avenues for research into the strategies in achieving effective cancer prevention.

### Potential Relationship between TMAO and Cancer: What More Should We Know?

With recent studies started showing evidence on a possible relationship between TMAO and cancer, it would be of great interest that this relationship is to be investigated further, which would help establish whether there is a causal role of TMAO in cancer. As TMA/TMAO production is primarily dependent on the intestinal bacteria, such investigation would enable us to explore and potentially generate new avenues of research into, and a better understanding of, the inter-relationship between the intestinal microbiota and cancer. This would thereby be useful in providing useful clues for the development of alternative therapeutic strategies in cancer prevention or treatment, through the manipulation of intestinal microbiota. In this section, the authors set out certain areas of research work that should be useful in shedding further light on the link between the intestinal microbiota, TMAO and cancer.

### Further exploration of the molecular mechanisms of TMAO-associated carcinogenesis

As indicated above, despite the availability of data indicating a positive association between TMAO and cancer risks, there are currently very limited studies that investigate the molecular basis of this association. Indeed, Xu *et al* 25 had suggested a number of genetic pathways that could be involved in the relationship between TMAO and CRC. Although previous studies provided evidence that inflammation could be a potential mechanism linking TMAO and carcinogenesis, current data provide little indication on how TMAO-associated pathways, such as cell cycle progression and Wnt signalling, could contribute to cancer from the molecular perspective. Moreover, most studies investigating the potential mechanisms of TMAO-induced carcinogenesis was conducted by cell treatment with TMAO, which do not enable the investigation of the effect of physiological production of TMAO on the proposed mechanisms. More research efforts should therefore be directed towards the exploration, through both *in vitro* and *in vivo* studies, of the changes in the activities of the pathways that are implicated in carcinogenesis upon TMAO production, rather than TMAO treatment. For example, transcriptomic analyses of colon cells extracted from mice fed with a diet high in TMA precursors (including choline and carnitine) would enable a further understanding on the changes in gene expression associated with increased TMAO production. Expression proteomic techniques may also be utilised to assess the changes in the expression level of these genes at the protein level, to confirm the data obtained from transcriptomic analyses. This would provide useful clues as to whether molecular and cellular pathways known to be implicated in carcinogenesis could be affected by TMAO. Thereafter, pathway analyses may be employed to explore further the pathways that may be involved in TMAO-associated carcinogenesis, based on the genes identified to have exhibited changes in expression level upon the intake of a high-choline or high-carnitine diet. Such studies would complement the data from studies involving cellular TMAO treatment, enhancing the evidence from studies that investigate the molecular basis on how TMAO is related to CRC and other cancers.

### Could diet-induced changes in the composition of intestinal microbiota be linked to cancer risks through modulation of TMA/TMAO production?

Previous studies suggested an effect of diet on the composition of intestinal microbiota 75. With the discovery that both the compositional changes of intestinal microbiota and TMAO are associated with cancer risk, it is hypothesised that TMA and TMAO production could be a factor that bridges the relationship between diet, intestinal microbiota and cancer. Consistent with this, vegetarian subjects were found to possess a different intestinal microbiota composition and exhibit a reduced level of TMAO production compared to omnivorous subjects 11, strengthening the evidence that diet could play a role in the modification of the composition of intestinal microbiota and TMAO production. However, to date, limited studies have been performed to investigate whether diet could be linked to cancer risks via changes in the level of intestinal TMA/TMAO production. Therefore, studies that investigate whether frequent intake of functional foods known to cause a health-promoting change in the composition of intestinal microbiota, such as rice bran 76, 77, could modulate plasma TMAO levels would be of value. Such studies would provide evidence as to whether alterations in TMA, and the subsequent TMAO, production could serve as a link between alterations in intestinal microbiota and carcinogenesis.

Indeed, previous research demonstrated that the intake of soy products, a functional food consumed widely in Asia, would lead to an alteration in intestinal microbiota 78 and a lowered cancer risk 79. Following these lines of evidence, it would be of interest to investigate whether soy product consumption would confer a lowered cancer risk through shaping the intestinal microbiota that results in lowered TMA/TMAO production. In light of this, dietary interventions with soy products, such as soymilk, may be performed in certain mouse models that mimic human CRC, such as *CDX2P 9.5-NLS lac*Z transgenic mice previously developed and used by Hinoi *et al* 80, and confirm whether soymilk consumption in these mice would lead to a reduced plasma TMAO levels, together with a modification of their intestinal microbiota and a reduction in the number of tumours present. Similarly, with the finding that vegetarian diets could lead to alterations in the composition of the gut microbiota and a concomitant reduction in TMAO production 11, studies on the effect of vegetarian diets in these mice on tumour numbers may also be of value. With the inhibitory effect of such dietary interventions on tumour formation established in mice, further studies involving such dietary interventions can be implemented among cancer patients, assessing whether they would confer any protective effects against carcinogenesis in humans. After the intervention, plasma levels of TMAO and cancer-associated biomarkers, such as Carcinoembryonic Antigen (CEA) 81, 82, should be determined via blood tests, coupled with an assessment of the changes of the composition of the intestinal microbiota of the intervention participants. These assessments would help evaluate whether the intervention has an effect on a concomitant reduction of TMAO and CEA levels, through modulation of the intestinal microbiota. Such studies would be useful in providing mechanistic insights into how functional food intake could be linked to reduced cancer risks via the modification of the composition of intestinal microbiota, and thus providing further evidence that TMAO is related to cancer risks. Further, as there are no published interventional studies that investigate whether physiological changes in TMAO levels would lead to molecular events associated with carcinogenesis, the suggested research work set out above would provide a starting point to assess whether modulation of TMAO production could have a direct contribution to carcinogenesis.

### Could TMAO be related to other cancers too?

While previous studies demonstrate a potential association between TMAO and colorectal and prostate cancers, it would also be of interest to investigate whether TMAO is associated with higher risks of other cancer types as well. Previous studies indicated that the changes in the composition of the microbiome not only could be associated with increased risk of CRC, but also that of breast 83 and gastric cancers 84. Indeed, these two cancer types were also postulated to be among the TMAO-related diseases by Xu *et al* 25, although epidemiological data that support this finding are lacking. Nevertheless, these findings suggest that TMAO could also be associated with both breast and gastric cancers. Further metabolomic profiling studies are therefore required to establish whether a correlation exists between TMAO levels and risk of these cancers. Such studies would provide further evidence for the potential link between TMAO and carcinogenesis, and would open up further avenues for the research into the molecular aspects of this link.

## Concluding Remarks

The past decade revealed an increase in the number of studies demonstrating the involvement of the intestinal microbiota in carcinogenesis, and previous research had also indicated a role of diet in the modulation of the composition of intestinal microbiota. However, the molecular basis of how diet-induced changes in intestinal microbiota are related to cancer is still not fully understood. The discovery that TMAO could potentially be associated with the risk of certain cancers, and that they can be produced by the intestinal bacteria from TMA precursors ingested in diet, has certainly provided us with opportunities to explore further on the relationship between the intestinal microbiota and cancer. Future research efforts should be directed towards the unravelling of the molecular mechanisms and pathways leading to TMAO-induced carcinogenesis through *in vivo* and *in vitro* studies. Further investigations on whether intake of health-promoting functional foods known to modify the composition of intestinal microbiota can lead to modifications in TMA and TMAO production levels would also be useful in deciphering the upstream events in diet-induced cancers such as CRC. These studies would ultimately enable us to gain a better understanding on the previously unappreciated relationship between TMAO and cancer. More importantly, they would inform the molecular basis on how changes in the composition of the intestinal microbiota could contribute to carcinogenesis, and whether consumption of certain functional foods that modify the intestinal microbiota, such as rice bran or soy products, would be effective in cancer prevention and intestinal health promotion.

## Abbreviations

CEA: Carcinoembryonic Antigen
CRC: colorectal cancer
ER: endoplasmic reticulum
FMO3: flavin-containing monooxygenase 3
IL-6: interleukin-6
NLRP3: nod-like receptor family pyrin domain containing 3
NMR: nuclear magnetic resonance
ROS: reactive oxygen species
TMA: trimethylamine
TMAO: trimethylamine-N-oxide
TNF-α: tumour necrosis factor-alpha

## References

[1] Torre LA, Bray F, Siegel RL (2015). Global cancer statistics, 2012. CA Cancer J Clin.

[2] Borges-Canha M, Portela-Cidade JP, Dinis-Ribeiro M (2015). Role of colonic microbiota in colorectal carcinogenesis: a systematic review. Rev Esp Enferm Dig.

[3] Gagnière J, Raisch J, Veziant J (2016). Gut microbiota imbalance and colorectal cancer. World J Gastroenterol.

[4] Wu S, Rhee KJ, Albesiano E (2009). A human colonic commensal promotes colon tumorigenesis via activation of T helper type 17 T cell responses. Nat Med.

[5] Cuevas-Ramos G, Petit CR, Marcq I (2010). Escherichia coli induces DNA damage in vivo and triggers genomic instability in mammalian cells. Proc Natl Acad Sci U S A.

[6] Sheflin AM, Whitney AK, Weir TL (2014). Cancer-promoting effects of microbial dysbiosis. Curr Oncol Rep.

[7] Hullar MA, Burnett-Hartman AN, Lampe JW (2014). Gut microbes, diet, and cancer. Cancer Treat Res.

[8] Demarquoy J, Georges B, Rigault C (2004). Radioisotopic determination of L-carnitine content in foods commonly eaten in Western countries. Food Chem.

[9] Zeisel SH, da Costa KA (2009). Choline: an essential nutrient for public health. Nutr Rev.

[10] Zhu Y, Jameson E, Crosatti M (2014). Carnitine metabolism to trimethylamine by an unusual Rieske-type oxygenase from human microbiota. Proc Natl Acad Sci U S A.

[11] Koeth RA, Wang Z, Levison BS (2013). Intestinal microbiota metabolism of L-carnitine, a nutrient in red meat, promotes atherosclerosis. Nat Med.

[12] Tang WH, Wang Z, Levison BS (2013). Intestinal microbial metabolism of phosphatidylcholine and cardiovascular risk. N Engl J Med.

[13] Wang Z, Klipfell E, Bennett BJ (2011). Gut flora metabolism of phosphatidylcholine promotes cardiovascular disease. Nature.

[14] Romano KA, Vivas EI, Amador-Noguez D (2015). Intestinal microbiota composition modulates choline bioavailability from diet and accumulation of the proatherogenic metabolite trimethylamine-N-oxide. MBio.

[15] Craciun S, Balskus EP (2012). Microbial conversion of choline to trimethylamine requires a glycyl radical enzyme. Proc Natl Acad Sci U S A.

[16] Falony G, Vieira-Silva S, Raes J (2015). Microbiology Meets Big Data: The Case of Gut Microbiota-Derived Trimethylamine. Annu Rev Microbiol.

[17] Cho CE, Taesuwan S, Malysheva OV (2017). Trimethylamine-N-oxide (TMAO) response to animal source foods varies among healthy young men and is influenced by their gut microbiota composition: a randomized controlled trial. Mol Nutr Food Res.

[18] Cheung W, Keski-Rahkonen P, Assi N (2017). A metabolomic study of biomarkers of meat and fish intake. Am J Clin Nutr.

[19] Krüger R, Merz B, Rist MJ (2017). Associations of current diet with plasma and urine TMAO in the KarMeN study: direct and indirect contributions. Mol Nutr Food Res.

[20] Landfald B, Valeur J, Berstad A (2017). Microbial trimethylamine-N-oxide as a disease marker: something fishy?. Microb Ecol Health Dis.

[21] Rohrmann S, Linseisen J, Allenspach M (2016). Plasma Concentrations of Trimethylamine-N-oxide Are Directly Associated with Dairy Food Consumption and Low-Grade Inflammation in a German Adult Population. J Nutr.

[22] Bae S, Ulrich CM, Neuhouser ML (2014). Plasma choline metabolites and colorectal cancer risk in the Women's Health Initiative Observational Study. Cancer Res.

[23] Liu X, Liu H, Yuan C (2017). Preoperative serum TMAO level is a new prognostic marker for colorectal cancer. Biomark Med.

[24] Guertin KA, Li XS, Graubard BI (2017). Serum Trimethylamine N-oxide, Carnitine, Choline and Betaine in Relation to Colorectal Cancer Risk in the Alpha Tocopherol and Beta Carotene Study. Cancer Epidemiol Biomarkers Prev.

[25] Xu R, Wang Q, Li L (2015). A genome-wide systems analysis reveals strong link between colorectal cancer and trimethylamine N-oxide (TMAO), a gut microbial metabolite of dietary meat and fat. BMC Genomics.

[26] Mondul AM, Moore SC, Weinstein SJ (2015). Metabolomic analysis of prostate cancer risk in a prospective cohort: The alpha-tocolpherol, beta-carotene cancer prevention (ATBC) study. Int J Cancer.

[27] Richman EL, Kenfield SA, Stampfer MJ (2012). Choline intake and risk of lethal prostate cancer: incidence and survival. Am J Clin Nutr.

[28] Bag S, Banerjee DR, Basak A (2015). NMR ((1)H and (13)C) based signatures of abnormal choline metabolism in oral squamous cell carcinoma with no prominent Warburg effect. Biochem Biophys Res Commun.

[29] Yu XF, Zou J, Dong J (2014). Fish consumption and risk of gastrointestinal cancers: a meta-analysis of cohort studies. World J Gastroenterol.

[30] Lee JY, Sim TB, Lee JE (2017). Chemopreventive and Chemotherapeutic Effects of Fish Oil derived Omega-3 Polyunsaturated Fatty Acids on Colon Carcinogenesis. Clin Nutr Res.

[31] Cho CE, Caudill MA (2017). Trimethylamine-N-Oxide: Friend, Foe, or Simply Caught in the Cross-Fire?. Trends Endocrinol Metab.

[32] Ufnal M, Pham K (2017). The gut-blood barrier permeability - A new marker in cardiovascular and metabolic diseases?. Med Hypotheses.

[33] Saweirs WM, Andrews DJ, Low-Beer TS (1985). The double sugar test of intestinal permeability in the elderly. Age Ageing.

[34] Jaworska K, Huc T, Samborowska E (2017). Hypertension in rats is associated with an increased permeability of the colon to TMA, a gut bacteria metabolite. PLoS One.

[35] Korniluk A, Koper O, Kemona H (2017). From inflammation to cancer. Ir J Med Sci.

[36] Janakiram NB, Rao CV (2014). The role of inflammation in colon cancer. Adv Exp Med Biol.

[37] Wu D, Cao M, Peng J (2017). The effect of trimethylamine N-oxide on Helicobacter pylori-induced changes of immunoinflammatory genes expression in gastric epithelial cells. Int Immunopharmacol.

[38] Jung JJ, Noh S, Jeung HC (2010). Chemokine growth-regulated oncogene 1 as a putative biomarker for gastric cancer progression. Cancer Sci.

[39] Kumari N, Dwarakanath BS, Das A (2016). Role of interleukin-6 in cancer progression and therapeutic resistance. Tumour Biol.

[40] Wajant H (2009). The role of TNF in cancer. Results Probl Cell Differ.

[41] Cao Z, Fu B, Deng B (2014). Overexpression of Chemokine (C-X-C) ligand 1 (CXCL1) associated with tumor progression and poor prognosis in hepatocellular carcinoma. Cancer Cell Int.

[42] Chen ML, Zhu XH, Ran L (2017). Trimethylamine-N-Oxide Induces Vascular Inflammation by Activating the NLRP3 Inflammasome Through the SIRT3-SOD2-mtROS Signaling Pathway. J Am Heart Assoc.

[43] Yue C, Yang X, Li J (2017). Trimethylamine N-oxide prime NLRP3 inflammasome via inhibiting ATG16L1-induced autophagy in colonic epithelial cells. Biochem Biophys Res Commun.

[44] Huang CF, Chen L, Li YC (2017). NLRP3 inflammasome activation promotes inflammation-induced carcinogenesis in head and neck squamous cell carcinoma. J Exp Clin Cancer Res.

[45] Wang H, Luo Q, Feng X (2018). NLRP3 promotes tumor growth and metastasis in human oral squamous cell carcinoma. BMC Cancer.

[46] He Q, Fu Y, Tian D (2018). The contrasting roles of inflammasomes in cancer. Am J Cancer Res.

[47] Al-Obaide MAI, Singh R, Datta P (2017). Gut Microbiota-Dependent Trimethylamine-N-oxide and Serum Biomarkers in Patients with T2DM and Advanced CKD. J Clin Med.

[48] Grivennikov SI, Karin M (2011). Inflammatory cytokines in cancer: tumour necrosis factor and interleukin 6 take the stage. Ann Rheum Dis.

[49] Nowiński A, Ufnal M (2018). Trimethylamine N-oxide: A harmful, protective or diagnostic marker in lifestyle diseases?. Nutrition.

[50] Randrianarisoa E, Lehn-Stefan A, Wang X (2016). Relationship of Serum Trimethylamine N-Oxide (TMAO) Levels with early Atherosclerosis in Humans. Sci Rep.

[51] Missailidis C, Hällqvist J, Qureshi AR (2016). Serum Trimethylamine-N-Oxide Is Strongly Related to Renal Function and Predicts Outcome in Chronic Kidney Disease. PLoS One.

[52] Golia E, Limongelli G, Natale F (2014). Inflammation and cardiovascular disease: from pathogenesis to therapeutic target. Curr Atheroscler Rep.

[53] Bäck M, Yurdagul A Jr, Tabas I (2019). Inflammation and its resolution in atherosclerosis: mediators and therapeutic opportunities. Nat Rev Cardiol.

[54] Akchurin OM, Kaskel F (2015). Update on inflammation in chronic kidney disease. Blood Purif.

[55] Qi J, You T, Li J (2018). Circulating trimethylamine N-oxide and the risk of cardiovascular diseases: a systematic review and meta-analysis of 11 prospective cohort studies. J Cell Mol Med.

[56] Tang WH, Wang Z, Kennedy DJ (2015). Gut microbiota-dependent trimethylamine N-oxide (TMAO) pathway contributes to both development of renal insufficiency and mortality risk in chronic kidney disease. Circ Res.

[57] Seldin MM, Meng Y, Qi H (2016). Trimethylamine N-Oxide Promotes Vascular Inflammation Through Signaling of Mitogen-Activated Protein Kinase and Nuclear Factor-κB. J Am Heart Assoc.

[58] Sun G, Yin Z, Liu N (2017). Gut microbial metabolite TMAO contributes to renal dysfunction in a mouse model of diet-induced obesity. Biochem Biophys Res Commun.

[59] Huc T, Drapala A, Gawrys M (2018). Chronic, low-dose TMAO treatment reduces diastolic dysfunction and heart fibrosis in hypertensive rats. Am J Physiol Heart Circ Physiol.

[60] Collins HL, Drazul-Schrader D, Sulpizio AC (2016). L-Carnitine intake and high trimethylamine N-oxide plasma levels correlate with low aortic lesions in ApoE(-/-) transgenic mice expressing CETP. Atherosclerosis.

[61] Perumareddi P (2019). Prevention of Hypertension Related to Cardiovascular Disease. Prim Care.

[62] Frostegård J (2013). Immunity, atherosclerosis and cardiovascular disease. BMC Med.

[63] Reuter S, Gupta SC, Chaturvedi MM (2010). Oxidative stress, inflammation, and cancer: how are they linked?. Free Radic Biol Med.

[64] Federico A, Morgillo F, Tuccillo C (2007). Chronic inflammation and oxidative stress in human carcinogenesis. Int J Cancer.

[65] Li T, Chen Y, Gua C (2017). Elevated Circulating Trimethylamine N-Oxide Levels Contribute to Endothelial Dysfunction in Aged Rats through Vascular Inflammation and Oxidative Stress. Front Physiol.

[66] Oellgaard J, Winther SA, Hansen TS (2017). Trimethylamine N-oxide (TMAO) as a New Potential Therapeutic Target for Insulin Resistance and Cancer. Curr Pharm Des.

[67] Robbiano L, Mereto E, Corbu C (1996). DNA damage induced by seven N-nitroso compounds in primary cultures of human and rat kidney cells. Mutat Res.

[68] Bhat MY, Singh LR, Dar TA (2017). Trimethylamine N-oxide abolishes the chaperone activity of α-casein: an intrinsically disordered protein. Sci Rep.

[69] Bonuccelli G, Castello-Cros R, Capozza F (2012). The milk protein α-casein functions as a tumor suppressor via activation of STAT1 signaling, effectively preventing breast cancer tumor growth and metastasis. Cell Cycle.

[70] Gong B, Zhang LY, Pang CP (2009). Trimethylamine N-oxide alleviates the severe aggregation and ER stress caused by G98R alphaA-crystallin. Mol Vis.

[71] Lupachyk S, Watcho P, Stavniichuk R (2013). Endoplasmic reticulum stress plays a key role in the pathogenesis of diabetic peripheral neuropathy. Diabetes.

[72] Corazzari M, Gagliardi M, Fimia GM (2017). Endoplasmic Reticulum Stress, Unfolded Protein Response, and Cancer Cell Fate. Front Oncol.

[73] Yadav RK, Chae SW, Kim HR (2014). Endoplasmic reticulum stress and cancer. J Cancer Prev.

[74] Schonthal AH (2012). Targeting endoplasmic reticulum stress for cancer therapy. Front Biosci (Schol Ed).

[75] Chan CW, Wong RS, Law PT (2016). Environmental Factors Associated with Altered Gut Microbiota in Children with Eczema: A Systematic Review. Int J Mol Sci.

[76] Sheflin AM, Borresen EC, Wdowik MJ (2015). Pilot dietary intervention with heat-stabilized rice bran modulates stool microbiota and metabolites in healthy adults. Nutrients.

[77] So WK, Law BM, Law PT (2016). Current Hypothesis for the Relationship between Dietary Rice Bran Intake, the Intestinal Microbiota and Colorectal Cancer Prevention. Nutrients.

[78] Fernandez-Raudales D, Hoeflinger JL, Bringe NA (2012). Consumption of different soymilk formulations differentially affects the gut microbiomes of overweight and obese men. Gut Microbes.

[79] Khan SA, Chatterton RT, Michel N (2012). Soy isoflavone supplementation for breast cancer risk reduction: a randomized phase II trial. Cancer Prev Res (Phila).

[80] Hinoi T, Akyol A, Theisen BK (2007). Mouse model of colonic adenoma-carcinoma progression based on somatic Apc inactivation. Cancer Res.

[81] Locker GY, Hamilton S, Harris J (2006). ASCO 2006 update of recommendations for the use of tumor markers in gastrointestinal cancer. J Clin Oncol.

[82] Gonzalez-Pons M, Cruz-Correa M (2015). Colorectal Cancer Biomarkers: Where Are We Now?. Biomed Res Int.

[83] Xuan C, Shamonki JM, Chung A (2014). Microbial dysbiosis is associated with human breast cancer. PLoS One.

[84] Brawner KM, Morrow CD, Smith PD (2014). Gastric microbiome and gastric cancer. Cancer J.

